# FRAT1 Enhances the Proliferation and Tumorigenesis of CD133^+^Nestin^+^ Glioma Stem Cells In Vitro and In Vivo

**DOI:** 10.7150/jca.37622

**Published:** 2020-02-10

**Authors:** Geng Guo, Jing Liu, Yeqing Ren, Xinggang Mao, Yining Hao, Chengliang Zhong, Xiaolin Chen, Xiaogang Wang, Yongqiang Wu, Shizhong Lian, Lin Mei, Yuanli Zhao

**Affiliations:** 1 Department of Neurosurgery, The First Hospital, Shanxi Medical University, No.85 Jiefangnan Road, Taiyuan, Shanxi 030001, People's Republic of China; 2 Department of Neurosurgery, Xijing Hospital, Fourth Military Medical University, No.15 Changlexi Road, Xi'an, Shaanxi 710032, People's Republic of China; 3 GCP Center, The First Teaching Hospital of Tianjin University of Traditional Chinese Medicine, No.88 Changling Road, Tianjin 300192, People's Republic of China; 4 Department of Neurosurgery, Beijing Tiantan Hospital, Capital Medical University, No.6 Tiantan Xili, Beijing 100050, People's Republic of China; 5 Department of Orthopedics, The First Hospital, Shanxi Medical University, No.85 Jiefangnan Road, Taiyuan, Shanxi 030001, People's Republic of China

**Keywords:** Glioma stem cells (GSCs), Proliferation, The frequently rearranged in advanced T cell lymphomas-1(FRAT1), Wnt/β-catenin pathway

## Abstract

Glioma stem cells (GSCs) are considered the source for development, recurrence, and poor prognosis of glioma, so treatment targeted GSCs is of great interest. The frequently rearranged in advanced T cell lymphomas-1 (FRAT1) gene is an important member of the Wnt/β-catenin signaling transduction pathway, and aberrantly activation of Wnt signaling has been identified to contribute to the tumorigenesis, proliferation, invasion of a variety kinds of cancer stem cells. However, correlations between FRAT1 and GSCs and the specific mechanisms remain unclear. In this study, we aimed to investigate the effect of FRAT1 on GSCs proliferation, colony formation, sphere formation and tumorigenesity in vitro and in vivo and its underlying mechanism. Lentiviral transfection was used to construct GSCs with low FRAT1 expression. The expression of FRAT1 on GSCs proliferation in vitro was assessed by cell counting kit-8(CCK-8). Colony formation and sphere formation assays were conducted to assess the colony and sphere formation ability of GSCs. Then, an intracranial glioma nude mouse model was built to measure the effect of low FRAT1 expression on GSCs proliferation and tumorigenesity in vivo. Real-time PCR, Western blot, and Immunohistochemistry were processed to detect the mRNA and protein expressions of FRAT1, β-catenin in the glioma tissue of xenograft mice to study their correlations. The functional assays verifed that low FRAT1 expression inhibited CD133^+^Nestin^+^ GSCs proliferation, colony formation, sphere formation ability in vitro. In vivo GSCs xenograft mice model showed that low FRAT1 expression suppressed the proliferation and tumorigenesity of CD133^+^Nestin^+^ GSCs and reduced β-catenin mRNA and protein expression. Furthermore, the expression of FRAT1 and β-catenin were positively correlated. Altogether, results indicate that FRAT1 enhances the proliferation, colony formation, sphere formation and tumorigenesity of CD133^+^Nestin^+^ glioma stem cells in vitro and in vivo as well as the expression of β-catenin. Therefore, inhibiting proliferation of GSCs and FRAT1 may be a molecular target to GSCs in treating human glioma in the future.

## Introduction

Glioma is the most common primary malignant tumor of the adult central nervous system. Although existing glioma treatments (surgery, radiotherapy, and chemotherapy) have been improved, therapeutic efficacies have not been significantly enhanced 1. Increasing studies have confirmed the existence of a small subpopulation of cancer stem cells (CSCs) in hierarchically organized solid tumors, which are considered responsible for tumor initiation, proliferation, progression and metastasis 2, 3. Previous work has indicated the presence of CSCs in numerous human malignancies including glioma, which are known as glioma stem cells (GSCs) 4-10, and stem cell features found in GSCs, like self-renewing, unlimited proliferating, and multi-directional differentiated tumor primitive cells 11, are thought to contribute to the incidence, recurrence, and poor prognosis of glioma 12. Recently, molecular targeted therapy has become an important adjunct to glioma treatment and targeted therapy of GSCs is of interest.

Specific cell surface markers have been used in collectively identifying and isolating GSCs. CD133, a stem cell-specific marker found in several types of tumor, was also identified in increasing the tumorigenic ability and resistance to chemotherapy and radiotherapy of GSCs 13. Nestin, another important cell surface marker for GSCs, was found in increasing robust CSC properties, for instance, tumorsphere-forming ability and tumorsphere size 14. CD133 and nestin expressions in glioma have been found correlate increasingly with malignancy grades, and Nestin/CD133 co-expression has been thought to be an important feature of GSCs 13, 15, 16.

The Wnt/β-catenin signal transduction pathway is a highly conservative signaling pathway, and its abnormal activation is closely related to over-proliferation of glioma. Our studies previously indicated that an aberrantly activated Wnt signaling pathway existed in glioma 17, 18. Researches have delineated multiple transcription regulation mechanisms of downstream genes of Wnt pathway, which explained the tumorigenesis, proliferation, invasion and other stem cell properties maintained in CSCs through Wnt signaling 19-22, and recently, vital roles of Wnt pathway in modulating GSCs has also been identified 23.

The frequently rearranged in advanced T cell lymphomas-1 (FRAT1) proto-oncogene is an important member of the Wnt/β-catenin signaling transduction pathway. FRAT1 downregulation inhibits the proliferation, migration, and invasiveness of glioma cells. High FRAT1 expression in human glioma upregulates intracellular accumulation of β-catenin, suggesting that FRAT1 may promote the incidence and development of glioma through the Wnt/β-catenin signal transduction pathway 24, 25. Our previous study confirmed that high FRAT1 expression occurs in human glioma tissue and human glioma cell lines, and its expression is significantly associated with glioma malignancy. Given the high expression and protumorigenic role of FRAT1 in glioma, we investigated its function and molecular mechanism in GSCs biology. Here, we report that FRAT1 acts as a key regulator of β-catenin/Wnt signaling to modulate GSCs proliferation in vitro and in vivo. Our results are the first demonstration that the FRAT1 has the ability to activate the Wnt pathway in glioma stem cells to promote stemness and tumor progression.

## Materials and Methods

### Glioma cell culture

U251, U87 and SHG44 human glioma cell lines (purchased from the Cell Bank of Peking Union Medical College, Beijing, China) was cultured in DMEM complete medium (containing 10% fetal bovine serum, 100 U/ml penicillin, and 100 U/ml streptomycin) (Invitrogen, Carlsbad, CA) in a 37°C incubator with 5% CO_2_. Medium was changed daily and cells were passaged once every 2-3 days. PBS (Invitrogen) was used to wash cells, followed by enzymatic cell dissociation with 0.25% trypsin (Invitrogen). A slightly impaired brain tissue fragment of a patient who underwent intracerebral hemorrhage was used to culture primary astrocytes to be a control. Written informed consent was obtained from the patient. The grey matter of the brain tissue was dissociated, washed in PBS and dispersed repeatedly and then the resulting cell suspension was filtered and cultured in DMEM with 10% fetal bovine serum.

### Lentiviral transfection and collecting of stably transfected cells

Related sequence synthesis and plasmid packaging were supported by Genechem, Ltd., Shanghai, China, with the FRAT1 interference sequence of 5'-ACGCGGGTCCCAACCAGAA-3' and independent sequence of 5'-TTCTCCGAACGTGTCACGT-3'. First, 5×10^5^ cells/ml were seeded into 6-well plates and labeled as follows: U251-FRAT1-KD indicated U251 cells transfected with lentiviral vector containing FRAT1-interference to knock down FRAT1, U251-vector indicated U251 cells transfected with lentiviral vector containing an unrelated sequence and U251-control indicated normal U251 cells with PBS (equal volume of transfection reagents in U251-FRAT1-KD and U251-vector). Cells were incubated in a 37°C incubator with 5% CO_2_ for 18-24 h until 70-80% confluency, followed by transfection in accordance with the manufacturer's protocol (multiplicity of infection, MOI=5). 1 μg/ml puromycin (Sigma-Aldrich, St. Louis, MO, USA) was used to collect stably transfected U251 cells (U251-FRAT1-KD and U251-vector) for subsequent experiments.

### GSCs culture

Original culture media for all groups was replaced with serum-free stem cell culture media, including neurobasal (Invitrogen), 2% B-27 (Invitrogen), 20 ng/ml human EGF (Invitrogen), 20 ng/ml human bFGF (Invitrogen), and 2% glutamate (Invitrogen). One-half the media volume was changed every other day and cells were harvested after two weeks of incubation and labeled as: GSC-FRAT1-KD, GSC-vector and GSC-control.

### Flow cytometry for identification and sorting cultured GSCs

Accutase (Invitrogen) was used to fully dissipate cultured GSCs suspension, followed by placing 1×10^7^ GSCs in 100 μl PBS. Then, 10 μl mouse anti-human CD133-PRE monoclonal antibody (Miltenyi, BergischGladbach, Germany) and 10 μl rabbit anti-human nestin-FITC monoclonal antibody (Abcam, UK) were added. Cells were incubated in the dark at 4°C for 10 min. Incubated cells were washed in 2 ml PBS thoroughly to remove unbound antibody, followed by centrifugation, discarding of the supernatant and another incubation in 100 μl PBS on ice. Then cells were identified and sorted using flow cytometry. CD133^+^Nestin^+^ GSCs in all groups were collected for subsequent experiments.

### Immunofluorescent staining and identification of cultured GSCs

Immunofluorescent antibody staining was used to identify CD133, nestin, and FRAT1 expression in GSCs. Cultured GSCs were placed on glass slides coated with 40 g/l polylysine solution and post-fixed in 4% paraformaldehyde for 15 to 20 min, followed by washing three times in 0.01 mol/l PBS for 5 min each. After blocking non-specific antigen on the cell surface using 1% BSA at 37°C for 1 h, cells were incubated with 1:300 PBS-diluted primary antibody at 4°C for 8 h and 37°C for 1 h. Then, cells were incubated with 1:200 PBS-diluted fluorescent secondary antibody in the dark at 37°C for 40 min. Cell nuclei were eventually stained with DAPI solution (Abcam). After washing the stained cells with PBS, cytomorphology was observed with a fluorescent microscope and photographed with a digital camera. Primary antibodies used included rabbit anti-human CD133 monoclonal antibody (Abcam), rabbit anti-human nestin monoclonal antibody (Abcam), and mouse anti-human FRAT1 monoclonal antibody (Abcam). Secondary antibody used included Alexa Fluor 488 (goat anti-rabbit fluorescent antibody, Cell signaling technology, Danvers, MA) and Alexa Fluor 555 (goat anti-mouse fluorescent antibody, Cell signaling technology).

### Cell Counting Kit-8 (CCK-8) for viability detection of the in vitro cells

CCK-8 reagent (Dojindo, Japan) was used for measuring GSCs proliferation in accordance with the manufacturer's instructions. Cells in all groups were dissipated using Accutase, followed by seeding 4×10^3^ cells/100 μl cultured suspension into 96-well plates and the optical density (OD) was measured using an automated microplate reader on days 0 to 6 days (450 nm) wavelength. All assays were repeated three times.

### Colony formation assay

GSCs were seeded into 24-well plates (Corning) in serum-free stem cell culture medium, which contained neurobasal (Invitrogen), 2% B-27 (Invitrogen), 20 ng/ml human EGF (Invitrogen), 20 ng/ml human bFGF (Invitrogen), and 2% glutamate (Invitrogen), supplemented with 0.8% methyl cellulose. The medium was replenished every 5 days. The cells were fixed with 4% paraformaldehyde and then stained with 0.1% crystal violet crystal violet (Sigma-Aldrich) and counted after culturing for 14 days. A cluster larger than 50 mm in diameter was identified as a colony.

### Spheroid-formation assay

GSCs were cultured in serum-free stem cell culture containing neurobasal (Invitrogen), 2% B-27 (Invitrogen), 20 ng/ml human EGF (Invitrogen), 20 ng/ml human bFGF (Invitrogen), and 2% glutamate (Invitrogen) in 24-well (Corning). The spheres were counted at day 14 after being planted.

### Intracranial xenograft models and magnetic resonance imaging (MRI)

All animal experiments were approved by the local animal experimental ethics committee and were conducted according to the NIH Guide for the Care and Use of Laboratory Animals. Four- to six-week-old specific-pathogen-free (SPF) BALB/c-nu male mice (purchased from Beijing HFK Bioscience, Ltd., Beijing, China, 16-18g) were randomly divided into groups with three mice per group. Nude mice were anesthetized with isoflurane gas and fixed in a small animal stereotactic apparatus (KOPF940). An intracranial glioma nude mouse model was established according to a previously reported experimental method 26 by collecting approximately 1×10^5^ cells/ 5 μl GSCs suspension from each group (GSC-FRAT1-KD, GSC-vector and GSC-control) and injecting the cell suspension into intracranial caudate nucleus of all mice. The mice were labeled as follows: mice-FRAT1-KD, mice-vector and mice-control. After modeling intracranial xenograft mice, 7.0 T MRI scanner (BrukerBioSpin, Billerica, MA, USA) was used to obtain intracranial tumor images, followed by measuring the maximum anteroposterior diameter (L), transverse diameter (W), and height (H) of tumors using a RadiAnt DICOM Viewer to calculate tumor volume as follows: V = π/6×L×W×H (mm^3^).

### Real-time quantitative PCR (RT-qPCR)

Total RNA of GSCs and xenograft mice gliomas was extracted using the Trizol reagent (Tiangen, China) in accordance with the manufacturer's instructions. PrimeScriptTM II 1^st^Strand cDNA Synthesis kit (TaKaRa, Dalian, China) was used for cDNA reverse transcription. cDNA products were used as DNA templates, and GAPDH was used as internal reference for real-time PCR using 7500 Fast Real-time PCR System (ABI, Waltham, MA) and SYBR Premix Ex Taq II (TaKaRa). Real-time PCR primers used were designed by TaKaRa as follows: Upstream FRAT1: 5'-GCCCTGTCTAAAGTGTATTTTCAG-3', downstream FRAT1: 5'-CGCTTGAGTAGGACTGCAGAG-3'; upstream β-catenin: 5'-ACTGGCAGCAACAGTCTTACC-3', downstream β-catenin 5'-TTTGAAGGCAGTCTGTCGTAAT-3'; upstream internal reference GAPDH: 5'-GAAGTGAAGGTCGGAGTCA-3' and downstream internal reference GAPDH: 5'-TTCACACCCATGACGAACAT-3'. Relative gene expression data were analyzed using the 2^-ΔΔCt^ method. This experiment was repeated three times.

### Western blot analysis

GSCs and intracranial glioma tissues collected from all groups of xenograft mice were homogenized in cell lysis buffer, followed by centrifugation to collect supernatant. Extracted total protein from each sample was quantified with a BCA Protein Assay Kit (Pierce, Rockford, IL). Forty micrograms of total protein from each sample was separated via 12% SDS-PAGE gel, followed by transfer to nitrocellulose membranes (Millipore, Bedford, MA). Membranes were blocked with non-specific antigen in 5% skin milk at room temperate for 2 h. After PBS washing, the protein membrane was incubated with 1:200 mouse anti-human FRAT1 monoclonal antibody, 1:1,000 mouse anti-human β-catenin monoclonal antibody, and 1:1,000 rabbit anti-human β-tubulin monoclonal antibody (Abcam) overnight at 4°C in a shaker. After three washes in PBS washing, the blot was incubated with relevant HRP-labelled secondary antibodies (1:1,000 goat anti-mouse secondary antibody or 1:1,000 goat anti-rabbit secondary antibody) on a shaker at room temperature for 2 h. After washing, ECL chemiluminescent reagent was used for blot development. Then, Scion Image software (Scion Corporation, Frederick, MD) was used to calculate the average OD of each protein band. Ratios of gray value of target bands (FRAT1 and β-catenin) and the β-tubulin band were used to quantify relative expression of target proteins. This experiment was repeated three times.

### Immunohistochemistry

Intracranial gliomas of all mouse groups were dissected and post-fixed in 10% formalin, followed by conventional paraffin-embedding, tissue sectioning into 4 µm thickness, and mounting to polylysine coated slides. After high-pressure heat retrieval of antigens, each tissue section was quenched in 3% H_2_O_2_ and washed in PBS for three times, followed by blocking antigen with non-immune serum for 30 min and incubating tissue sections with 1:50 mouse anti-human FRAT1 monoclonal antibody and 1:1000 mouse anti-human β-catenin monoclonal antibody (Abcam) at 4°C overnight. After three PBS washes, tissue sections were incubated with secondary antibody at 37°C for 30 min and developed in DAB solution (Sigma-Aldrich), followed by rinsing in distilled water, hematoxylin nuclear-staining, and neutral gum mounting.

### Histological assessment

Pathologists blinded to treatments read immunohistochemical data and positive FRAT1 and β-catenin staining was brownish-yellow. To evaluate the immunoreactivity score (IRS) of FRAT1 and β-catenin in glioma, protein expression was observed under 200× magnification to select the most obviously stained region. Five random high-power fields at 400× magnification were chosen and 1,000 cells were counted using an eyepiece mesh micrometer. With reference to a previous study, IRS of each stained section was assessed using semi-quantitative analysis. IRS is the percent of positively (PP) stained cells × staining intensity (SI) of each section. PP for each tissue section was categorized as follows: Score 0, <1% positive cells; Score 1, 1-25% positive cells; Score 2, 26-50% positive cells: Score 3, 51-75% positive cells; and Score 4, >75% positive cells. In addition, SI for each tissue section was categorized as follows: Score 0, no staining; Score 1, weak staining; Score 2, moderate staining; and Score 3, intensive staining. SI scores were based on SIs for most cells. The IRS of each section ranged from 1-12. An IRS of 0 represented negative results and IRS from 1-12 represented positive results 27.

### Statistical analysis

SPSS 19.0 (SPSS Inc., Chicago, IL) software was used for statistical analysis of experimental data, which are presented as means ± standard deviation. Comparisons of means of multiple groups were first performed using one-way ANOVA, followed by pairwise comparison of means between groups using student-Newman-Keuls test. Correlation between FRAT1 IRS and β-catenin IRS was analyzed using Pearson correlation analysis. p*<*0.05 was considered to be a statistically significant difference.

## Results

### The expression of FRAT1 was elevated in human malignant glioma cell lines

Our previous work has showed that compared with the normal human brain cells, FRAT1 was highly expressed in three high grade glioma-derived cell lines U251, U87 and SHG44. Finally, we chose U251 whose expression level of FRAT1 was the highest to establish in-vitro cell model 24.

### FRAT1 highly expressed in CD133^+^Nestin^+^ GSCs

**Figure 1:**
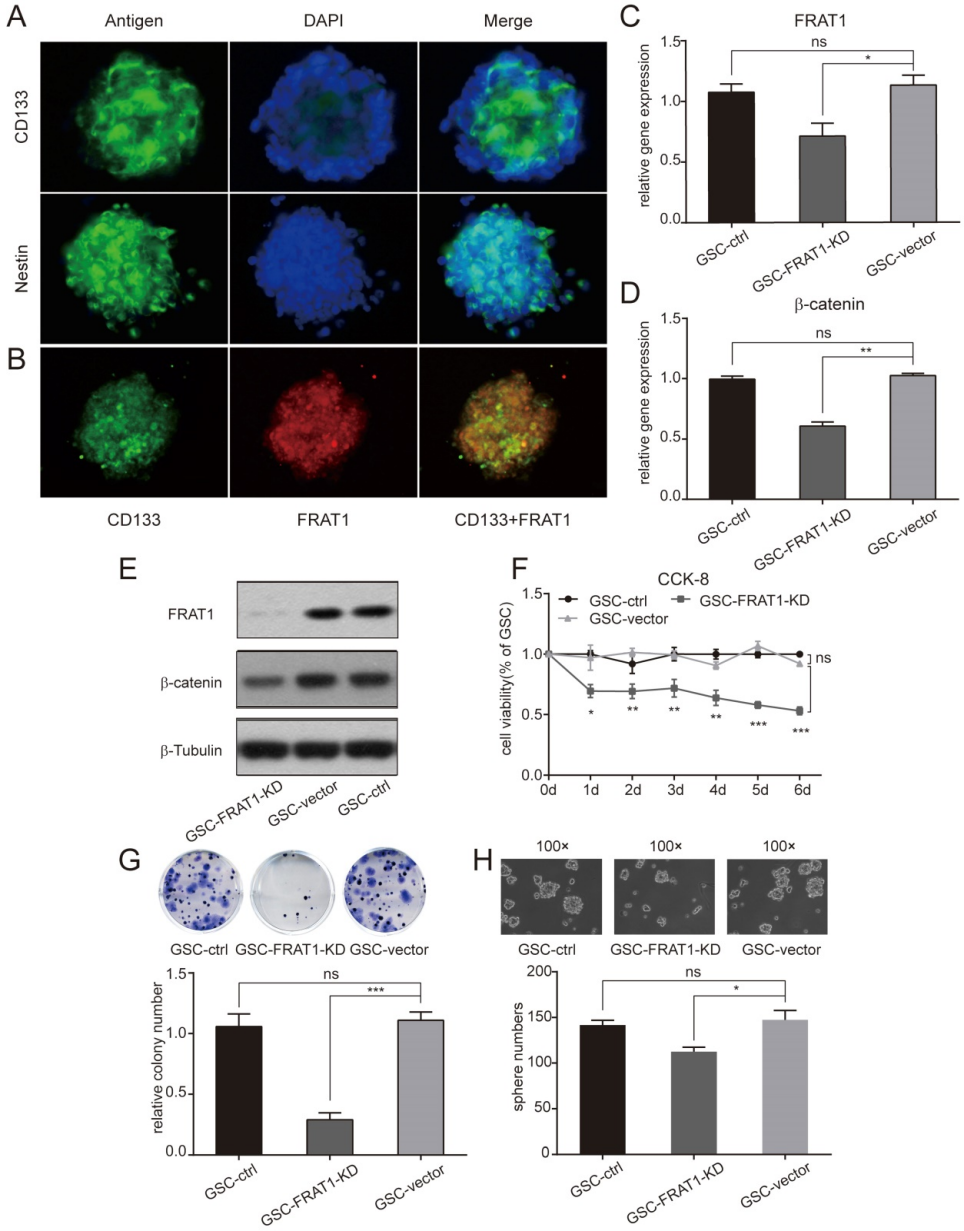
Immunofluorescent assay was performed and low expression of FRAT1 inhibited CD133^+^Nestin^+^ GSCs proliferation, colony and sphere formation ability in vitro. (A) Immunofluorescent assay to measure CD133 and nestin expression in GSCs (400×). Fluorescent microscopy for CD133-positive staining (green fluorescence) and nestin-positive staining (green fluorescence) in cytoplasm of GSCs; DAPI-positive staining (blue fluorescence) in nucleus of GSCs. (B) Immunofluorescent assay for FRAT1 and CD133 expression in GSCs (400×). Fluorescent microscopy for CD133-positive staining (green fluorescence) and FRAT1-positive staining (red fluorescence) in cytoplasm of GSCs. FRAT1 and CD133 proteins co-localized in GSCs. (C-D) Real-time PCR for mRNA expression of FRAT1 and β-catenin in GSCs in vitro. GAPDH was used as an internal reference. (E) Western blot of FRAT1 and β-catenin protein expression in GSCs in vitro. β-Tubulin was used as an internal reference. (F) CCK-8 assay detected the effect of low FRAT1 expression on the viability of in vitro GSCs. A450 OD of GSCs was continuous monitored from days 0 to 6. (G) Representative images of GSCs colony formation and the relative colony number of GSCs are shown. (H) Representative images of GSCs sphere formation are shown (100×) as well as the sphere number of GSCs. *p < 0.05, **p < 0.01; ***p < 0.001; ns, non-significant.

CD133^+^Nestin^+^ GSCs were identified with flow cytometry. The above collected GSCs were stained with stem cell antibodies (anti-CD133 and anti-nestin) and DAPI was used for nuclear staining. GSCs were positive for CD133 and nestin (Fig. 1A). Anti-FRAT1 antibody was used to confirm GSCs with CD133 expression, and cultured GSCs (GSC-vector and GSC-control) co-expressed FRAT1 and CD133 (Fig. 1B).

### Establishment of CD133^+^ Nestin^+^GSCs with stable inhibition of FRAT1 gene

Stably transfected U251 cells (U251-FRAT1-KD and U251-vector) were collected and cultured GSCs (GSC-FRAT1-KD, GSC-vector and GSC-control) were labeled with anti-CD133 and anti-nestin and CD133^+^Nestin^+^ GSCs were identified with flow cytometry. RT-qPCR was performed and the results showed that there was a significant reduction in FRAT1 and β-catenin mRNA levels in GSC-FRAT1-KD cells compared to GSC-vector (p<0.05, p<0.01, respectively). In addition, there was no significant difference in mRNA levels between GSC-vector and GSC-control for both FRAT1 and β-catenin (Fig. 1C and D). Western blot analysis also showed reduced protein levels of FRAT1 and β-catenin in GSC-FRAT1-KD cells (Fig. 1E). The results together confirmed the successful construction of low FRAT1 expression CD133^+^ Nestin^+^ GSCs.

### Low FRAT1 expression inhibited GSCs proliferation, colony formation and sphere formation ability

GSCs proliferation ability was tested by CCK-8 and figure 1F shows the growth curves of all groups from day zero to day six. OD for GSC-FRAT1-KD was significantly lower than that for GSC-vector (p<0.05 at day 1; p<0.01 at day 2, day 3, day 4; p<0.001 at day 5, day 6). There was no significant difference in OD between GSC-vector and GSC-control. These results indicated that low FRAT1 expression inhibited GSCs proliferation in vitro (Fig. 1F). In addition, the colony formation assay showed that reduction in FRAT1 expression decreased the colony formation ability of GSCs. The relative colony number of GSC-FRAT1-KD was significantly reduced than that of GSC-vector (p<0.001) and there was no obvious difference between GSC-vector and GSC-control (Fig. 1G). Sphere formation assay was also performed and the sphere number of GSC-FRAT1-KD was shown to be significantly decreased compared to GSC-vector (p<0.05), while there was no significant difference between GSC-vector and GSC-control (Fig. 1H).

### Low FRAT1 expression inhibited in vivo GSCs proliferation

**Figure 2:**
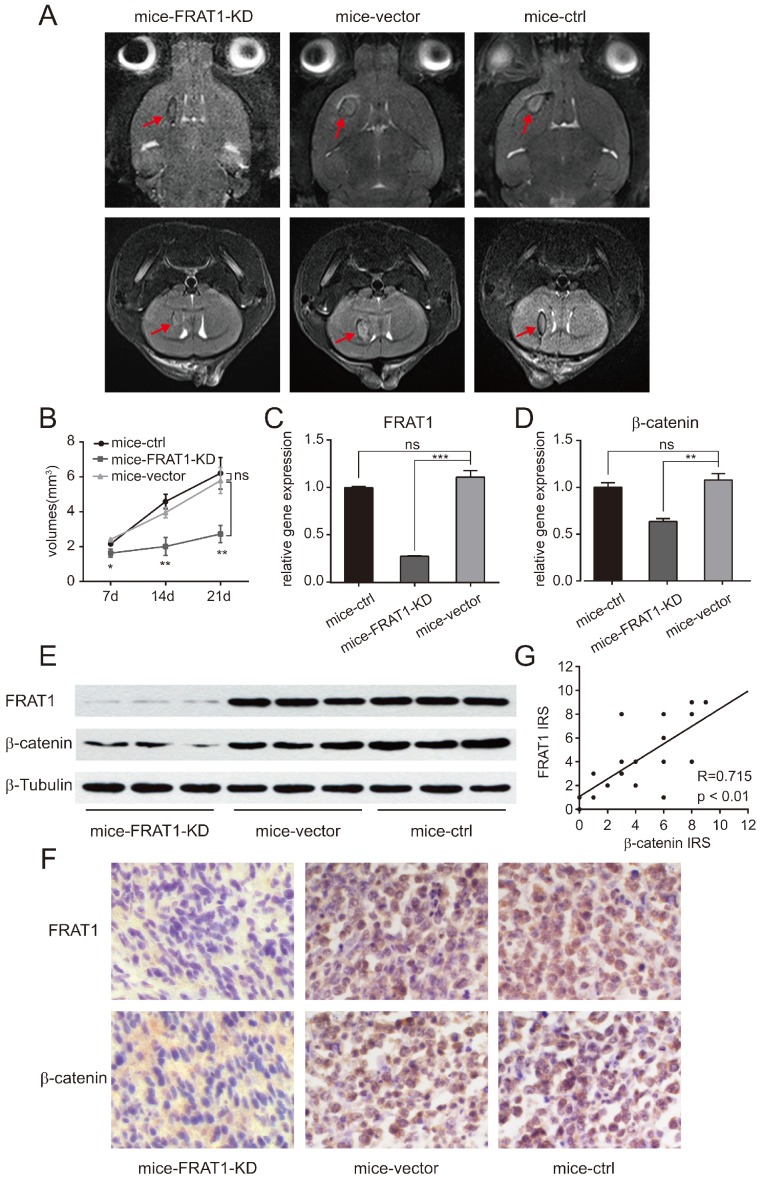
Low expression of FRAT1 inhibited GSCs proliferation in vivo and also reduced the expression of β-catenin. (A) Small animal MRI for low FRAT1 expression and in vivo GSCs proliferation. MRI for axial and coronal intracranial gliomas (red arrows) of all groups of xenograft mice on day 21 after inoculation. (B) RadiAnt DICOM Viewer used to measure intracranial glioma volumes of all groups of xenograft mice on days 7, 14, and 21 after inoculation to prepare a growth curve for intracranial gliomas. (C-D) Real-time PCR for mRNA expression of FRAT1 and β-catenin in glioma tissues of xenograft mice. GAPDH was used as an internal reference. (E) Western blot of FRAT1 and β-catenin protein expression in glioma tissues of xenograft mice. β-Tubulin was used as an internal reference. (F) Representative images showing the immunohistochemistry of FRAT1 and β-catenin protein expression in glioma tissues of xenograft mice (×400). FRAT1-positive protein staining localized in the cytoplasm of glioma cells. β-catenin-positive protein staining localized in the cytoplasm and (or) nucleus of glioma cells. (G) Scatter plot of correlation between FRAT1 IRS and β-catenin IRS in the glioma tissues of xenograft mice. Pearson correlation analysis showed that there was a positive correlation between FRAT1 IRS and β-catenin IRS in the glioma tissues of xenograft mice. *p < 0.05, **p < 0.01; ***p < 0.001; ns, non-significant.

After establishing the intracranial glioma nude mouse model, small animal MRI was used to monitor growth of intracranial tumors and to measure intracranial tumor volume on days 7, 14, and 21. Intracranial tumor volumes of all groups of xenograft mice were increased over time, with most significant result on day 21 of modeling (Fig. 2A and B) The glioma volume of mice-FRAT1-KD was significantly smaller than mice-vector (p<0.05 at day 7; p<0.01 at day 14, day 21); whereas no significant difference in glioma volumes was found between mice-vector and mice-control (Fig. 2B). The results confirmed that low FRAT1 expression inhibited GSCs proliferation in vivo.

### Positive relationship between FRAT1 and β-catenin mRNA and protein expression in glioma tissues of the xenograft mice

Real-time PCR confirmed relative mRNA expression of FRAT1 and β-catenin in the glioma tissues of mice-FRAT1-KD were significantly lower than mice-vector respectively (p<0.001 for FRAT1, p<0.01 for β-catenin); but there was no significant difference in relative mRNA expression of FRAT1 and β-catenin in glioma tissues between mice-vector and mice-control (Fig. 2C and D). Western blot analysis also showed reduced protein levels of FRAT1 and β-catenin in glioma tissues of mice-FRAT1-KD (Fig. 2E). Immunohistochemical analysis demonstrated that FRAT1 and β-catenin protein staining intensitis for mice-FRAT1-KD were reduced, respectively (Fig. 2F). Pearson correlation analysis confirmed that FRAT1 IRS was positively associated with β-catenin IRS (Fig. 2G). Thus, glioma tissues of tumor-bearing mice with low FRAT1 mRNA and protein expression also had low in β-catenin mRNA and protein expression, suggesting a positive correlation between FRAT1 and β-catenin expression.

## Discussion

Glioma is common, heterogeneous intracranial primary malignancy with a median survival time of approximately 15 month 28. GSCs are a subpopulation of glioma cells with self-renewal, multipotency and tumor initiating ability, and are considered the primary cause of infinite proliferation, high invasion, postoperative recurrence, and multidrug resistance of glioma 29, 30. Accumulating evidences suggest that GSCs contribute to tumor recurrence and are the driving force behind glioma relapses. Studies have indicated that non-tumorigenic cancer cells with high heterogeneity could undergo reprogramming and become GSCs. So researchers have started to focus on GSCs as the “root cells” initiating malignancy 31.GSCs express GSC markers, such as CD133 and nestin 13, 14, 16, 30, 32 which were found increasing positively with glioma grades, and their prognostic significance has also been identified 16. Thus, identification and selectively clearing GSCs by specific cell markers may provide an essential target for future glioma therapy.

The Wnt/β-catenin signaling pathway is a highly conserved transduction pathway which serves as the main regulator of nervous system development. It is involved in great amounts of key process in the embryonic development of higher animals, such as cell proliferation, differentiation, apoptosis, and participates in a variety of stages in neural development, such as stem cell maintenance, proliferation, differentiation 33, 34. Our work has found that the expression of FRAT1 and β-catenin was reduced in glioma tissues of tumor-bearing mice and there was a positive correlation between FRAT1 and β-catenin protein expression, which suggested that low FRAT1 inhibited the expression of β-catenin. β-catenin dependent Wnt pathway is the canonical Wnt pathway which is believed to contribute to GSCs proliferation and maintenance of stem cell features. Abnormal activation of Wnt/β-catenin pathway is regarded as a characteristic of GSCs and has been shown to mediate resistance to therapy, potentially providing an opportunity for therapeutic targeting 35, 36. Gong's group 23 reported that the aberrant activation of Wnt/β-catenin pathway and expressions of stem cell marker nestin in GSCs to promote cell cycle transformation from the G1 to the S phase, leading to self-renewal and proliferation of GSCs. Jiang and colleagues 37 reported that increased β-catenin expression enhanced proliferation of glioma cells and inhibiting apoptosis of CD133^+^ human GSCs. Secreted frizzled-related protein (SFRP), a Wnt/β-catenin signaling pathway inhibitor, blocked the cell cycle as well as inhibiting tumor cell proliferation ad spheroid formation of GSCs 38. Pleiomorphic adenoma gene like-2 (PLAGL-2) was found to activate the Wnt/β-catenin pathway in GSCs and contributed to its self-renewal 39, 40. Human achaetescute homolog (ASCL1), a vital transcription factor involved in neuronal differentiation is also critical for GSCs maintenance and propagation via upstream regulation of the Wnt/β-catenin signaling pathway 41. In conclusion, Wnt/β-catenin signaling pathway plays an active role of in GSCs' characters as well as glioma biology behavoir.

The human FRAT1 gene is localized in the long arm of chromosome 10 (10q24.1). FRAT1 protein has a molecular weight of 29 kD, containing 279 amino acids. As an extensively recognized oncogene, FRAT1 is overexpressed in ovarian cancer 42, non-small cell lung cancer 43, pancreatic cancer 44, gastric cancer 45, and liver cancer 46, and it is associated with poor clinical prognosis. Disheveled (Dsh/Dvl) of the Wnt/β-catenin signaling pathway acts on FRAT1, allowing FRAT1 to compete with Axin to bind to the same site on glycogen synthase kinase-3β (GSK-3β), resulting in the separation of GSK-3β from the scaffold complex (mainly composed of APC, Axin, GSK-3β, and CK1), thereby inhibiting GSK-3β-mediated phosphorylation and degradation of β-catenin. This allows accumulation of β-catenin in the cytoplasm and translocation of β-catenin from the cytoplasm into nucleus. Nuclear β-catenin binds to T cell factor/lymphoid enhancer factor (TCF/LEF) to form a transcription complex, increasing the expression of proto-oncogenes, c-myc and cyclin D1, and leading to tumorigenesis 47. Our previous work has clarified the oncogenic role of FRAT1 in glioma. We have demonstrated that FRAT1 mRNA and protein are highly expressed in glioma tissues and glioma cell lines, with expression being elevated with increasing pathological glioma grade. FRAT1 expression is positively correlated with proliferation of glioma and seriously affects patient prognosis. In addition, downregulated FRAT1 expression inhibits the proliferation of glioma cells, induces cell cycle arrest in the G1 phase, and weakens subcutaneous tumor formation in nude mice 17, 18, 24, 25, 42. Similarly, Fan's group 46 reported that hypoxia significantly induces overexpression of FRAT1 gene in liver cancer tissues. Knocking out the FRAT1 gene downregulates β-catenin, cyclin D1, and c-myc expression in liver cancer cells and inhibits tumor cell proliferation by regulating the Wnt/β-catenin signaling pathway. Goksel and colleagues 48 revealed that FRAT1 expression is significantly upregulated in CD133^+^ CD44^+^ prostate cancer stem cells and FRAT1 overexpression activates Wnt/β-catenin signaling pathway by inducing β-catenin/TCF activity.

In the present study, we evaluated the effect of FRAT1 on GSCs proliferation in vitro and in vivo, and stably downregulated FRAT1 in U251 cells through lentiviral transfection, and induced GSCs culture in serum-free medium. Then, we sorted CD133^+^Nestin^+^ GSCs using flow cytometry. GSCs viability was assessed with a CCK-8 assay, confirming that low FRAT1 expression inhibited GSCs proliferation in vitro. Colony and sphere formation ability of GSCs were assessed by colony and sphere formation assays respectively, and downregulation of FRAT 1 inhibited GSCs stem-cell potential with decreased colony and sphere formation efficiency. Through small animal MRI to examine glioma growth in brains of xenograft nude mice, we confirmed that downregulated FRAT1 expression inhibited the GSCs proliferation in vivo. Subsequent applications of real-time quantitative PCR, Western blot, and immunohistochemistry to measure mRNA and protein expression of FRAT1, β-catenin in Wnt signaling transduction in glioma tissues of the tumor-bearing mice indicated that low FRAT1 mRNA and protein expressions in xenograft mice, accompanied with downregulated mRNA and protein expressions of β-catenin. In addition, a positive correlation was found between FRAT1 and β-catenin protein expression, suggesting that low FRAT1 expression inhibited β-catenin activity and also FRAT1 was likely to inhibit the GSCs functions such as proliferation, colony formation, sphere formation, and tumorigenesity through Wnt/β-catenin signaling pathway.

However, there is some limitation of this research. According to our previous work 24, the expression of FRAT1 was increased in human malignant glioma cell lines U251, U87 and SHG44 compared with the normal human brain cells, and in this research we chose U251 whose expression level of FRAT1 was the highest to establish in-vitro cell model. It would be more appropriate to utlize additional cell lines such as U87 and other tumor cell lines to evaluate the effect of FRAT1 on GSCs proliferation, colony formation, sphere formation in vitro and tumorigenesity in vivo.

The present study has shown that low FRAT1 expression inhibited CD133^+^Nestin^+^ GSCs proliferation and tumorigenesis in vitro and in vivo. Also a positive correlation was found between FRAT1 and β-catenin protein expression. Further investigation is necessary to define how FRAT1 regulates GSCs through Wnt/β-catenin signaling pathway and then change the characters of glioma. Our study confirms that FRAT1 provides vital functions in GSCs, which provides new insights into the role of FRAT1 in tumor progression and reinforces the relevance of FRAT1 and GSCs as viable therapeutic targets for glioma.

## Abbreviations

GSCs: Glioma stem cells
FRAT1: the frequently rearranged in advanced T cell lymphomas-1
CCK-8: Cell Counting Kit-8
MRI: magnetic resonance imaging
RT-qPCR: Real-time quantitative PCR
IRS: immunoreactivity score
PP: percent of positively
SI: staining intensity
TCF/LEF: T cell factor/lymphoid enhancer factor
GSK-3β: glycogen synthase kinase-3β
